# Novel Robotic Test Rig for Camshaft Geometry Measurement with a Collaborative Robot

**DOI:** 10.3390/s26072206

**Published:** 2026-04-02

**Authors:** Agnieszka Sękala, Jacek Królicki, Tomasz Blaszczyk, Piotr Ociepka, Krzysztof Foit, Gabriel Kost, Maciej Kaźmierczak, Grzegorz Gołda, Wojciech Jamrozik

**Affiliations:** 1 Faculty of Mechanical Engineering, Silesian University of Technology, Konarskiego 18A, 44-100 Gliwice, Poland; piotr.ociepka@polsl.pl (P.O.); gabriel.kost@polsl.pl (G.K.); maciej.kazmierczak@polsl.pl (M.K.); grzegorz.golda@polsl.pl (G.G.); wojciech.jamrozik@polsl.pl (W.J.); 2 Autorobotics Sp. z o.o., ul. Kozielska 16, 47-400 Racibórz, Poland; j.krolicki@autorobotics.pl; 3 IT Department, Zealand-University of Applied Sciences, Lyngvej 21, 4600 Køge, Denmark; tomb@zealand.dk

**Keywords:** robotic camshaft geometry measurement, comparative measurements, robotic ultrasonic measurement, quality control system, preventive quality control, collaborative robot-based quality control

## Abstract

**Highlights:**

**What are the main findings?**

- A robotic ultrasonic measurement approach enables the full measurement of the cam profile over the entire 0–360° rotation range.
- Validation against CMM reference measurements confirms repeatable profile measurements, while pointwise error metrics (mean difference, standard deviation of differences, and maximum absolute deviation) reveal a strong angular dependence associated with the local cam geometry.

**What are the implications of the main findings?**

- Ultrasonic measurement deviations are primarily driven by surface curvature and sensor incidence angle/orientation (geometry–acoustics interaction), rather than by purely random measurement noise.
- Measurement strategies that account for object geometry and employ multi-orientation sensor positioning can significantly improve the robustness of the method for industrial camshaft inspection.

**Abstract:**

This paper presents the design and experimental validation of an innovative robotic test stand for measuring camshaft cam geometry, intended to support preventive quality control in high-volume production. The proposed solution integrates a collaborative robot with a dedicated measurement setup to enable repeatable positioning of the inspected camshaft and automated acquisition of geometric features critical for functional performance. A complete measurement methodology was developed, including the measurement sequence, data acquisition procedure, and processing of the recorded signals to determine key cam geometry parameters. To verify the reliability of the proposed approach, measurement results obtained using the robotic stand were compared with reference data acquired using conventional metrology tools and standard inspection procedures. Experimental studies confirmed that the developed stand provides repeatable measurement results, enabling the stable identification of the examined geometric features across repeated trials. Moreover, a high level of agreement was observed between the measurement data obtained using the proposed method and the reference measurements, demonstrating the suitability of the cobot-based test stand for preventive quality control applications in industrial environments. The concept presented offers a scalable and flexible alternative to manual inspection and dedicated special-purpose gauges, with potential benefits in terms of inspection throughput and standardization of quality control workflows. The novelty of the approach lies in the indirect ultrasonic measurement model combined with a quadrant-based sensor orientation strategy and repeatable 90° camshaft indexing, enabling full-profile acquisition within the robot workspace.

## 1. Introduction

Geometrical measurements constitute one of the key elements of the machine-part manufacturing process, as they provide the data necessary to assess product conformity with specifications and to support decision-making within quality control systems. In high-volume production, preventive actions become particularly important. These include the early detection of deviations and emerging trends, enabling intervention at a stage when process correction is still feasible and when the costs associated with scrap and rework can still be minimized.

The development of automated and digitized manufacturing systems (Industry 4.0) is shifting the emphasis from off-line inspection toward in-line and on-machine measurement solutions. This transition is enabled by rapid data acquisition, integration with control systems, and near-real-time analysis. The literature highlights the growing importance of data fusion, automated interpretation of measurement results, and adaptive quality control systems that support flexible manufacturing and shorten inspection cycles [1]. Within this framework, the concept of Zero-Defect Manufacturing (ZDM) has gained particular relevance. ZDM treats quality assurance as a coherent set of strategies―including detection, prediction, and prevention―further strengthened by digitalization and Industry 4.0 technologies [2,3].

A particularly relevant example of a component whose geometry directly affects system performance of a system is the cam mechanism. In internal combustion engines, the camshaft and cam profile determine valve motion characteristics, including lift and the timing of valve opening and closing. These parameters directly influence the gas exchange process, overall engine efficiency, fuel consumption, and exhaust emissions. Studies on variable camshaft timing and variable valve timing strategies indicate that modifying valve timing significantly affects engine efficiency and environmental performance, thereby confirming the importance of accurate valvetrain geometry and the need for reliable inspection methods [4].

Cam geometry measurement remains a demanding task, primarily due to the need to ensure repeatable datum establishment, stable measurement conditions, and sufficient inspection throughput. Classical approaches, such as coordinate measuring machines (CMMs), provide high accuracy; however, they often require relatively long measurement times and greater operator involvement. The literature indicates that automated cam profile inspection using CMM systems can be time-consuming. As an alternative, dedicated inspection stations may be used. Such stations are typically designed for a specific geometry type and enable direct comparison of measured data with nominal design values [5].

This paper presents the concept and validation of a robotic test station for measuring camshaft cam geometry, intended to support preventive quality control activities in high-volume production. The primary objective is to assess the conformity of cam geometry with reference (nominal) data for preventive quality inspection rather then reverse engineering. A collaborative robot is employed to automate the measurement sequence and ensure repeatable positioning within the proposed measurement methodology.

In the proposed system, the collaborative robot supports the operator during rapid camshaft inspection by ensuring repeatable positioning of the ultrasonic measurement head, adjusting its orientation when necessary, and moving it between successive cam lobes. At the same time the operator mounts and indexes the camshaft and supervises the inspection process. At this stage, ultra-precise measurements are not essential because the camshaft is mounted directly by the operator and its manufacturing quality is commonly verified through preliminary visual inspection.

An important justification for using a collaborative robot is the safety concept it provides for human operators. Unlike conventional industrial robots, collaborative robotic systems can operate without dedicated physical safety fencing because they are designed to meet human–robot interaction safety requirements. This significantly improves the accessibility and usability of the measurement workstation while also reducing its spatial footprint.

The measurement results obtained on the robotic test station were compared with reference data acquired using conventional methods in a metrology laboratory (ZEISS, VAST XT). The experiments conducted confirmed the repeatability of the measurements and demonstrated high level of agreement between the results obtained using the proposed method and the reference measurements.

Despite extensive research on robotic metrology and dedicated cam inspection solutions, there remains a need for flexible, robot-assisted approaches that combine repeatable positioning with measurement models suitable for rapid preventive inspection under industrial conditions. This study addresses this gap by proposing and validating a robot-based ultrasonic measurement method for cam inspection in quality control applications.

The main contributions of this paper are as follows:

- The design of a cobot-based test rig for camshaft inspection;
- An indirect measurement model for $r_{i}\left(\gamma \right)$ based on ultrasonic distance measurements;
- A validation framework based on comparison with reference measurements obtained using a coordinate measuring machine (CMM) (ZEISS VAST XT).

The remainder of this paper is organized as follows. Section 1 (Introduction) presents the background and motivation for the study. Section 2 (Related Work) reviews the current state of the art and outlines the research motivation. Section 3 (Proposed Experimental Methodology) describes the developed test station and the measurement methodology. Section 4 (Test Rig Prototype for Camshaft Geometry Measurement) presents the prototype robotic test station used for measuring camshaft cam geometry. Section 5 (Comparative Analysis of Reference and Ultrasonic Measurements) reports the experimental results and compares them with reference measurement data. Finally, Section 7 (Conclusion) summarizes the results and provides concluding remarks.

## 2. Related Work

Quality management systems are founded on ensuring quality at every stage of production. From the manufacturing process perspective, a key factor is defining the inspection strategy, location, and frequency depending on the specific characteristics of the product. The main inspection strategies include off-line control, where the product is verified after the manufacturing process has been completed, and on-line control, which focuses on monitoring quality during process execution. For both strategies, implementation costs are of critical importance. Based on the literature on inspection planning and quality control economics [6,7,8], these costs can be grouped into three main categories:

- Processing costs―fixed and variable costs associated with producing specific goods;
- Inspection costs―fixed and variable costs related to performing quality inspection;
- Yield loss costs―losses resulting from defects generated during the production process.

These issues are directly addressed by Kang et al. [6], Tirkel and Rabinowitz [7], and Mittal and McNally [8], with the distinction that Kang et al. analyze processes involving human resources, whereas the remaining studies focus on inter-process automated quality control. The authors in [6,7,8] consistently emphasize that each quality inspection activity extends the production cycle and increases costs. Therefore, it is necessary to find a balance between the number of inspections and the cost components listed above while maintaining the required confidence level.

A related problem is discussed by Tzimerman and Herer [9], who note that manufacturing processes may be affected by random errors. These errors, together with the possibility of misclassifying product quality and the chosen inspection frequency, determine how quickly defective items can be detected. Their work aims to identify an optimal inspection policy that achieves a required confidence level for producing conforming products.

The development of the Industry 4.0 concept introduces new challenges for quality control and metrology, which must meet the requirements of automated and digitized manufacturing. Imkamp et al. [10] identify several key issues, including the transition from offline measurements toward measurements performed directly on the production line, such as in-line and on-machine approaches. They also emphasize real-time data fusion, in which metrology is embedded within networked control systems that analyze measurement data in cyber-physical system architectures. Furthermore, the automation of result interpretation through adaptive quality control systems is becoming increasingly important.

Adaptive quality control systems are systems in which measurement data acquired during production are automatically interpreted and used to adjust inspection or process control parameters in response to changing operating conditions. Their characteristic features include data integration, automated decision support, and feedback mechanisms enabling rapid reaction to detected deviations.

These issues extend earlier ideas formulated by Berthold et al. [11], summarized under the objectives of “faster, safer, more accurate, and more flexible,” which encompass:

- Reducing measurement time in manufacturing processes through automation of measurement procedures and system integration;
- Increasing the reliability of metrological systems;
- Improving measurement precision to meet increasingly stringent quality requirements;
- Adapting to variable production conditions, such as product personalization or small-batch manufacturing, which aligns directly with the Industry 4.0 paradigm.

A major challenge in contemporary manufacturing is Zero-Defect Manufacturing (ZDM). Psarommatis et al. [12] note that short production runs and product personalization, which are characteristic features of Industry 4.0, increase process complexity and consequently the risk of defects. This trend necessitates a shift in quality assurance strategies, including the adoption of tools based on artificial intelligence, the Internet of Things (IoT), and advanced data analytics. The goal is to eliminate defects during manufacturing by applying four interrelated strategies: detection, repair, prediction, and prevention.

Detection plays a pivotal role because the data acquired at this stage support corrective actions, including repair, and enable the development and training of predictive algorithms that facilitate defect prevention. The authors emphasize that ZDM should not be viewed as a single technology or procedure but rather as a philosophy that integrates multiple strategies into a holistic approach to manufacturing.

Powell et al. [13] argue that ZDM aligns with a zero-waste manufacturing strategy. Beyond the four core strategies, non-destructive inspection methods and the implementation of circular solutions, such as recycling and remanufacturing, can reduce material losses. In their view, ZDM can act as a catalyst for sustainable development by supporting the transition from a linear to a circular production model. Furthermore, the authors argue that the need for repairs, which consume additional resources and energy, can be reduced through effective prediction and prevention mechanisms, downstream error compensation, or selective part matching during assembly. Within the ZDM approach, it is therefore essential not only to acquire information about defects, but also to disseminate this knowledge throughout the production system. This can be facilitated by industrial information systems capable of integrating and distributing measurement data.

Industrial robots are currently integral to a wide range of manufacturing processes. They are most frequently used in operations related to material handling, assembly, and finishing tasks such as painting or polishing. Robotic workstations are often equipped with vision systems that support the control system in locating and recognizing parts as well as in decision-making.

In addition to conventional fixed vision systems, cameras and sensors mounted on robot manipulators are also widely used for detecting and identifying objects within the robot workspace. Kinnel et al. [14] describe a more advanced system in which 3D cameras are used to identify the position and orientation of objects with complex geometry. In this approach, the robot acts as a carrier that moves the camera to determine the most suitable viewpoint, enabling the acquisition of a large number of measurements points for reliable pose estimation.

Rao et al. [15] employ a fringe projector and a camera mounted on a robot arm to scan complex-shaped objects and generate a point cloud, which is subsequently used to create a computer-aided design (CAD) model. The authors also highlight the influence of robot positioning accuracy on the quality of the obtained results.

A different approach was proposed by Kiraci et al. [16], who mounted a laser distance sensor on a robot arm to measure an automotive body-in-white stage. The measurement results were reported to fall within the tolerance limits specified for typical body inspection while also demonstrating shorter measurement times compared to conventional coordinate measuring machine systems.

An analysis of current trends in implementing quality assurance procedures in manufacturing and adopting Industry 4.0 principles [17,18] motivated this study on the use of a robotic measurement system to inspect the geometry of an internal combustion engine camshaft. Developing an effective solution for automated robot-assisted evaluation of geometric parameters supports the transition toward measurements performed directly on the production line. Furthermore, collecting measurement data in electronic form, with the possibility of further transmission and processing, aligns directly with the Zero-Defect Manufacturing philosophy.

The reviewed studies indicate a clear trend toward in-line and on-machine metrology and the increased automation of inspection tasks. However, in the case of camshaft inspection, existing solutions remain either highly accurate but time-consuming (CMM-based inspection) or dedicated to specific geometries. Therefore, a flexible robotic test rig with a well-defined measurement model and an industrially feasible workflow is required.

## 3. Proposed Experimental Methodology

The proposed measurement procedure is based on the indirect determination of the cam radius $r_{i}(\gamma )$ from the ultrasonic distance signal d(γ), acquired at discrete angular positions γ over the full rotation range. Prior to the scanning stage, the geometric constant $R_{c}$ is identified using a dedicated measurement at $\gamma \;=\;90^{\circ }$, where the measured radius corresponds to the known base-circle radius, enabling analytical back-calculation of $R_{c}$.

To mitigate angle-dependent ultrasonic reflection effects, data the acquisition is performed over two angular intervals with two predefined sensor orientations (quadrant-based strategy). For each angular position, repeated measurements are collected to quantify repeatability and measurement uncertainty. The estimated cam profiles are subsequently validated against reference data obtained using a coordinate measuring machine (CMM), applying standard profile agreement metrics, including the mean difference, standard deviation of differences, and maximum absolute deviation.

**Figure 1:**
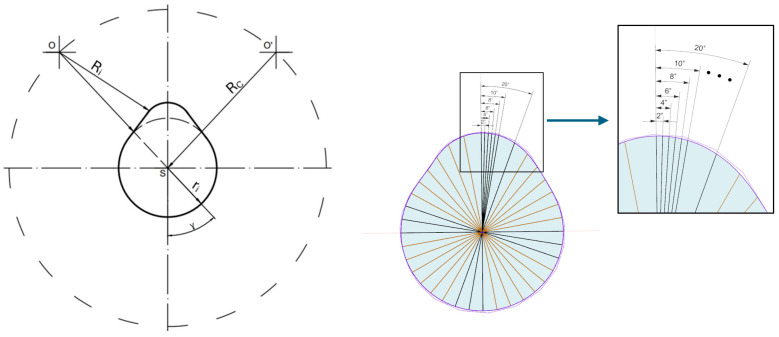
Proposed measurement method.

The output of the measurement process is the cam radius $r_{i}$, determined along the entire cam profile. Measurements are performed at angular positions γ, referenced to the camshaft rotation center S, starting from the initial measurement point O, with an angular 2° over a full 360° rotation (Figure 1).

To investigate the cam geometry of the examined component, an indirect measurement method was adopted. In this approach, the measuring system first performs direct measurements of auxiliary quantities, and the obtained results are subsequently processed using appropriate mathematical operations to determine the desired parameter.

(1) $$X\;=\;F(X_{1},\;X_{2},\;\ldots ,\;X_{m}),\;$$

where:

X―the value of the output parameter;

$X_{1},\;X_{2},\;\ldots ,\;X_{m}$―the values of auxiliary quantities, measured directly.

In accordance with (1), an algorithm was developed to determine the target output value $r_{i}$ based on known inputs. These includes the quantity $R_{i}$, defined as the distance measured from the initial measurement point to the cam surface, and the constant $R_{c}$, defined as the fixed distance from the initial point O (or $O^{\prime }$) to the center of the cam (i.e., the camshaft rotation axis). Based on these assumptions, an equation was derived that contains a single unknown.

(2) $$X\;=\;r_{i}\;=\;f\left(R_{i}\right)\;=\;Rc\;-\;R_{i},$$

where:

$i\;=\;\gamma \;\in \left(0{}^{\circ},\;2{}^{\circ},\;4{}^{\circ},\;6{}^{\circ},\;\ldots ,\;358{}^{\circ},\;360{}^{\circ}\right);$

$r_{i}$―cam radius r(γ) as a function of the angular position γ;

Rc―distance from the initial measurement point to the cam rotation axis;

$R_{i}$―distance from the initial measurement point to the cam plane as a function of the rotation angle *γ*;

*γ*―camshaft rotation angle.

The target measurement uncertainty was set to 0.1 mm, which is consistent with the intended preventive inspection purpose and the expected tolerance range of the examined features.

In this study, the angle γ represents camshaft indexing through rotation in the bearing supports, while the robotic manipulator is used solely for positioning the measurement head.

## 4. Test Rig Prototype for Camshaft Geometry Measurement

**Figure 2:**
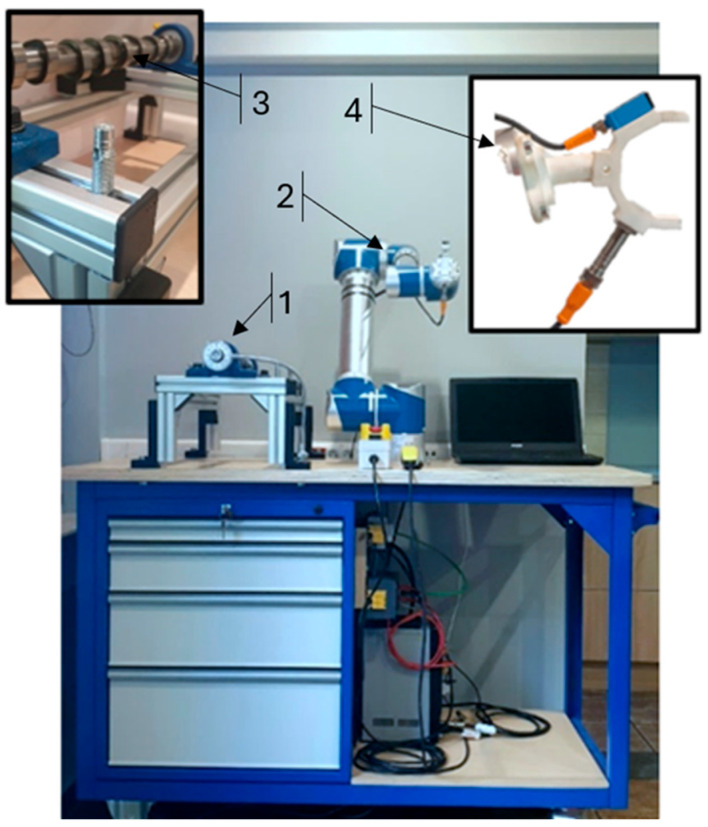
Robotic measurement station: (**1**) test object mounting fixture; (**2**) manipulator arm; (**3**) mounted camshaft; (**4**) developed measurement head.

A prototype robotic test rig was developed for camshaft cam geometry measurement. The system is centered on a Global ZEUS ZRA-0515 (ZEUS Co., Ltd., Hwaseong, Republic of Korea) collaborative robot, which is responsible for executing the measurement sequence and ensuring repeatable positioning and guidance of the measurement system components relative to the examined object. The design assumptions, functional structure, and measurement workflow are presented below. A photograph of the experimental setup is shown in Figure 2.

**Figure 3:**
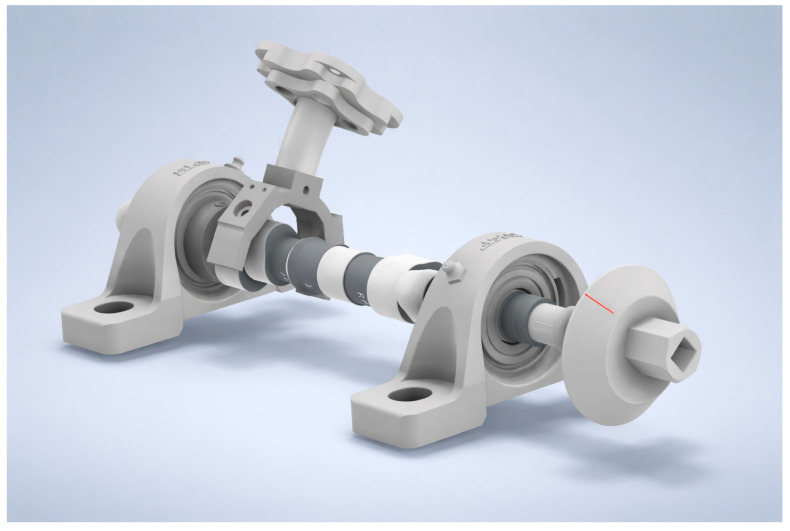
3D model of the assembly station.

The test object mounting fixture (1) was designed to securely hold the examined component, i.e., the camshaft, and to maintain a fixed and stable position of the workpiece throughout the measurement process. The camshaft is supported by two self-aligning bearings mounted in SKF (Göteborg, Germany) plummer block housings. The fixture is additionally equipped with a Jonnesway AG010118A (1/2 in) torque-wrench angle gauge (Jonnesway, Taipei, Taiwan). This gauge includes a flexible stem with a magnetic tip, allowing the angular dial to be stabilized in a precisely defined position. The angle gauge was used to ensure repeatable camshaft angular indexing during the measurements, with subsequent setups performed in 2° steps. The gauge was installed on the upper surface of the bolt head fastening the bearing housing to the aluminum profile. Mounting the magnet at alternative locations was not feasible due to the predominantly aluminum construction of the fixture. A 3D model of the mounting fixture is presented in Figure 3.

To determine the cam radius as a function of the camshaft rotation angle, the robotic station is used to experimentally measure the distance between the front face of the ultrasonic sensor head and a point located on the rotation axis of the examined component. The measurement is performed using a dedicated tool mounted on the robot end-effector.

**Figure 4:**
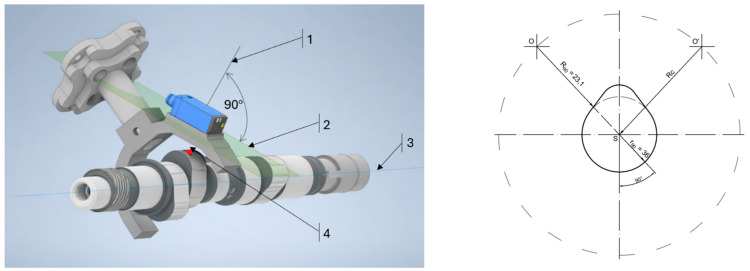
Principle of the measurement station design: 1―Measurement axis; 2―Sensor measuring head plane; 3―Camshaft rotation axis; 4―Point of maximum cam lift.

According to the developed measurement methodology, this distance corresponds to the parameter $R_{c}$, which is determined analytically by rearranging Equation (1). To obtain a reliable value of $R_{c}$, an experimental measurement was conducted on the robotic test rig at a camshaft rotation angle of $90^{\circ }$ (Figure 4). The zero position of the camshaft was defined such that the maximum cam lift point is aligned with the sensor measurement axis, with the axis oriented perpendicular to the sensor face.

It should be noted that the γ =90° position is achieved by camshaft rotation within the bearing supports (mechanical indexing), rather than by robot motion. This step serves as a reference (calibration-like) procedure used to determine $R_{c}$ based on the known value of $R_{90}$.

The experimental measurement performed at a camshaft rotation angle of $90^{\circ }$ enables the determination of the known parameter $R_{90}$, as this position corresponds to the constant base-circle radius of the cam (Figure 4).

Based on the general form of Equation (2), the equation was rearranged to determine the value of $R_{C}$ by substituting the values of the corresponding parameters.

(3) $$R_{C}\;=\;R_{i}\;+\;r_{i}\;=\;41.1\;mm,$$

where:

i =γ =90°;

$R_{90}\;=\;23.1\;mm;$

$r_{90}\;=\;18\;mm$.

Determining the value of $R_{c}$ enables the cam radius of the examined component to be calculated over the full rotation range in accordance with the developed measurement methodology. The output value of the parameter $R_{i}$ is computed automatically during measurement execution by the implemented algorithm.

**Figure 5:**
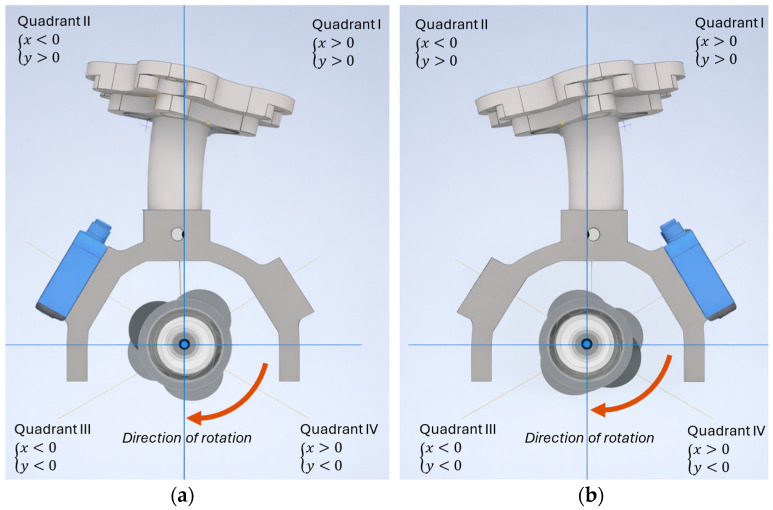
Graphical illustration of the proposed measurement strategy, where: (**a**) The first stage over the range from $2^{\circ }$ to $180^{\circ }$; (**b**) The second stage, performed over the range from $182^{\circ }$ to $360^{\circ }$.

To avoid issues related to the ultrasonic wave reflection angle from the cam surface, which directly affects measurement accuracy within specific angular ranges, the measurement procedure was divided into two stages for each cam. The first stage covers the angular range from $2^{\circ }$ to $180^{\circ }$, with the ultrasonic sensor oriented in the second quadrant of the coordinate system (Figure 5a). In the second stage, performed over the range from $182^{\circ }$ to $360^{\circ }$, the robot tool is rotated about its final rotational axis so that the sensor is positioned in the first quadrant (Figure 5b).

## 5. Comparative Analysis of Reference and Ultrasonic Measurements

**Figure 6:**
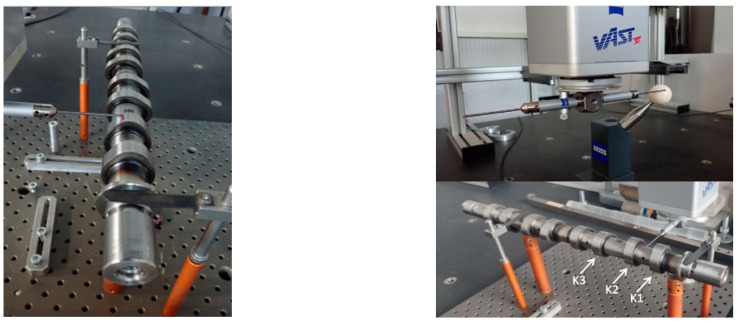
Camshaft mounted on the ZEISS ACCURA 7 MASS (Carl Zeiss, Germany) coordinate measuring machine (CMM).

**Figure 7:**
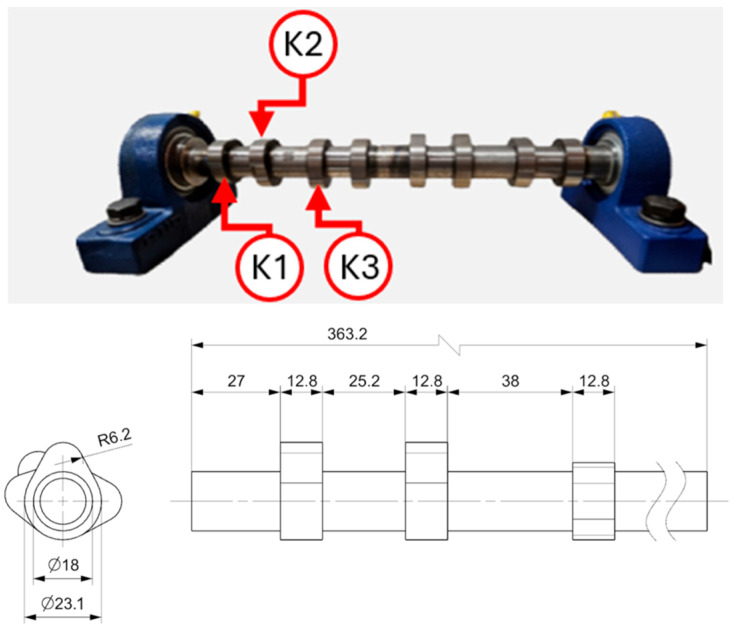
Camshaft mounted on bearing supports, with the first three cam lobes (K1, K2, and K3) selected for measurement.

Reference and ultrasonic measurement results were compared to evaluate the accuracy and repeatability of the proposed approach. The tested object is a Renault OEM camshaft (part number 82 00 100 527), shown in Figure 6. The first three measured cam lobes, denoted as K1, K2, and K3, are presented in Figure 7.

To quantitatively assess the consistency and compatibility of the cam lobe profiles obtained using the reference and alternative measurement systems, a set of standard comparative metrics was employed. These metrics are widely used in measurement data analysis and measurement system validation.

The comparative analysis considered: (a) a reference measurement method based on a coordinate measuring machine (CMM), providing high precision (ZEISS ACCURA 7 MASS; measurement range: 900 × 1200 × 700 mm); and (b) an alternative measurement method employing an ultrasonic proximity sensor (Sick UC4-1334B).

The technical specifications of the ZEISS ACCURA 7 MASS include: (a) length measurement error according to ISO 10360-2: MPEE = (1.6 + L/333) µm for a temperature range of 18–22 °C, or MPEE = (2.1 + L/300) µm for a temperature range of 18–26 °C; (b) probing system error according to ISO 10360-2: MPEP = 1.7 µm; (c) scanning probing error according to ISO 10360-4: MPETPH = 2.5 µm (test duration: 50 s); and (d) roundness deviation measurement uncertainty according to ISO 12181: MPERONt = 1.7 µm.

**Figure 8:**
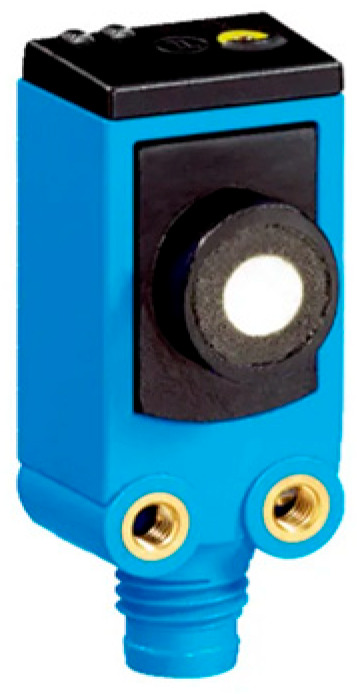
Ultrasonic distance sensor Sick UC4-1334B (SICK AG, Düsseldorf, Germany).

For the ultrasonic sensor SICK UC4-1334B, shown in Figure 8, measurements were conducted under controlled environmental conditions: (a) ambient temperature: 20.5 °C; and (b) relative air humidity: 50%. The measurement resolution of the ultrasonic sensor is 0.1 mm.

When comparing geometric features, it should be noted that cam lobe profiles can be constructed using different geometric definitions, e.g., harmonic, tangential, and concave. In this study, a tangential cam profile was used.

**Figure 9:**
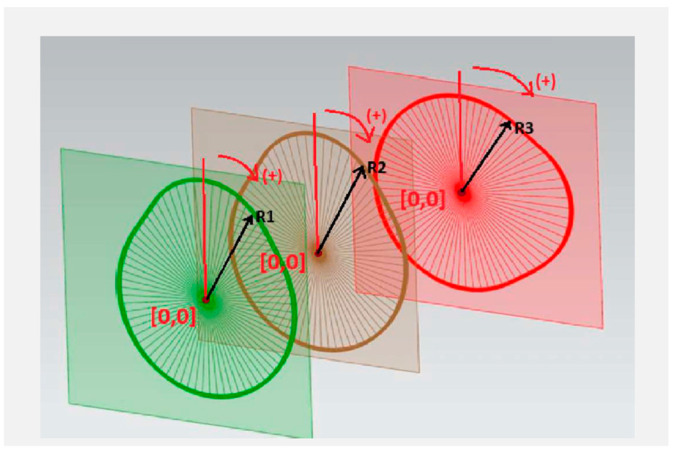
Measurement geometry and angular sampling scheme applied to each cam lobe.

The cam lobe radius was selected as the geometric parameter for comparison. For each of the three cam lobes, the radius was measured at angular intervals of 2° over the full rotational range from 0° to 360°. At each angular position, the measurement was repeated 50 times to assess repeatability and estimate measurement uncertainty. Figure 9 illustrates the measurement geometry and the angular sampling scheme applied to each cam lobe.

**Figure 10:**
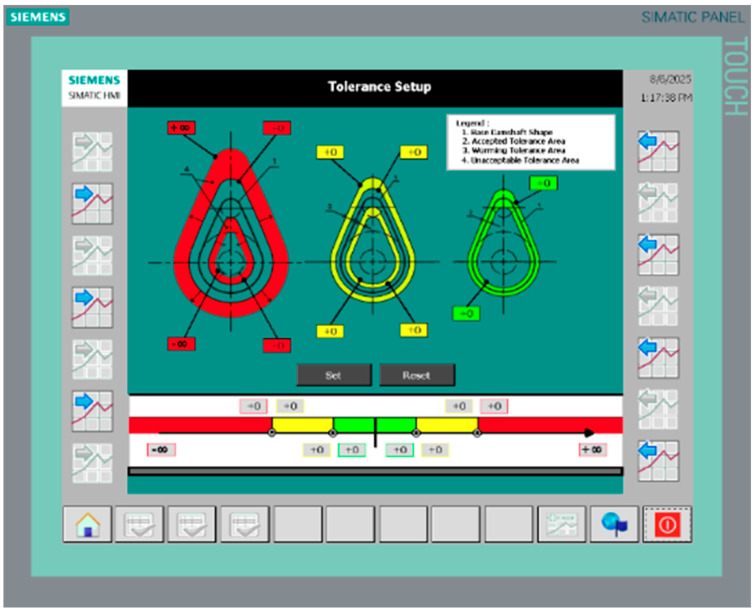
HMI panel showing the definition of measurement tolerance ranges.

Measurement data in tabular form, derived from the camshaft cam lobe geometry measurement protocol, were recorded using the alternative measurement method based on the ultrasonic displacement sensor SICK UC4-1334B. In addition, measurement tolerance ranges were defined in the human–machine interface (HMI), enabling classification of the measured values according to predefined alarm thresholds: (a) “Accepted Tolerance Area,” (b) “Warning Tolerance Area,” and (c) “Unacceptable Tolerance Area,” as shown in Figure 10. The classification criterion is the deviation of the measured value from the nominal (reference) value relative to the predefined tolerance thresholds specified in the HMI.

For each point of the cam lobe profile corresponding to a defined angular position, 50 repeated measurements were performed. This measurement strategy follows established industrial guidelines for the assessment of measurement process capability, which emphasize repeatability analysis and the evaluation of local, geometry-dependent effects rather than relying solely on global performance indicators [19]. Based on these repetitions, the mean radius value (${x¯}_{i})$ was determined for each angular position according to:

(4) $${x¯}_{i}=\;\frac{1}{n}\;\sum_{j\;=\;1}^{n}x_{ij},$$

where:

i = 1, …, m;

$x_{ij}$―radius measured at the i-th angular position (profile point) during the j-th repetition;

n = 50―number of measurement repetitions;

*m*―total number of angular sampling positions (e.g., 180 a 2° step over 360°).

The sample standard deviation ($s_{i})$ at each angular position *i* was determined as:

(5) $$s_{i}=\;\sqrt{\frac{1}{n\;-\;1}}\;\sum_{j\;=\;1}^{n}{\left(x_{ij}-{\bar{x}}_{i}\right)}^{2}$$

where:

i = 1, …, m;

$s_{i}$―sample standard deviation of the measured radius at angular position *i*;

*n* = 50―number of measurement repetitions;

*m*―total number of angular sampling positions (e.g., 180 for 2° step over 360°);

$x_{ij}$―the *j*-th measured radius at angular position *i*;

${x¯}_{i}$―the mean radius value at angular position *i*, computed from the n = 50 repetitions.

These parameters enable the assessment of measurement data dispersion and the stability of the recorded measurements for each applied measurement system.

After evaluating repeatability and stability, a comparative analysis between the ultrasonic and reference (CMM) measurements was performed. The first comparative metric applied was the mean profile radius difference (6), which characterizes the systematic offset between the profiles obtained using the two measurement methods. The quantity ${\bar{\Delta }}_{i}$ represents the local systematic deviation between the ultrasonic and reference measurements at a given angular position, obtained by averaging multiple ultrasonic measurement repetitions.

(6) $${\bar{\Delta }}_{i}=\frac{1}{n}\;\sum_{j=1}^{n}\left(r_{US,ij}\;-\;r_{CMM,i}\right)$$

where:

${\bar{\Delta }}_{i}$―mean profile radius difference at angular position *i*, representing the local systematic offset between the ultrasonic (US) and reference (CMM) measurements;

$r_{US,ij}$―radius value obtained from the ultrasonic measurement at the i-th angular position for the j-th repetition;

$r_{CMM,i}$―reference radius value obtained from the CMM at the same i-th angular position;

j = 1, 2, …, n―index of repeated ultrasonic measurements;

i = 1, 2, …, m―index of the angular sampling point along the cam lobe profile;

*n* = 50―the number of measurement repetitions;

*m*―total number of angular sampling positions (e.g., 180 for 2° step over 360°).

**Figure 11:**
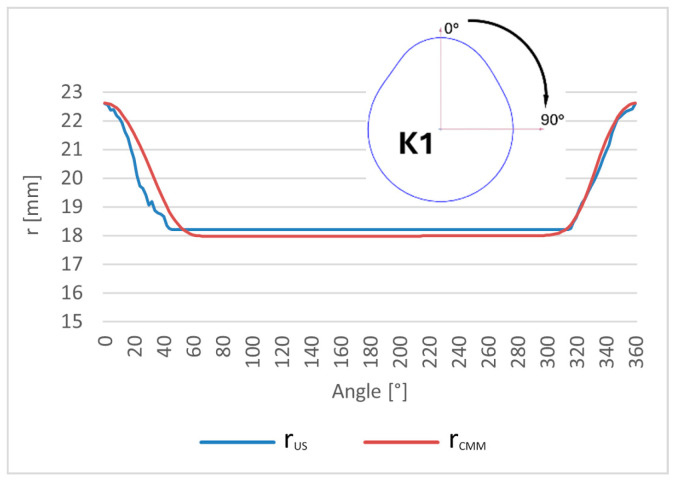
Comparison of the mean ${\bar{\Delta }}_{i}$ cam lobe K1 radius values measured by the robot with the reference values obtained from the coordinate measuring machine (CMM).

**Figure 12:**
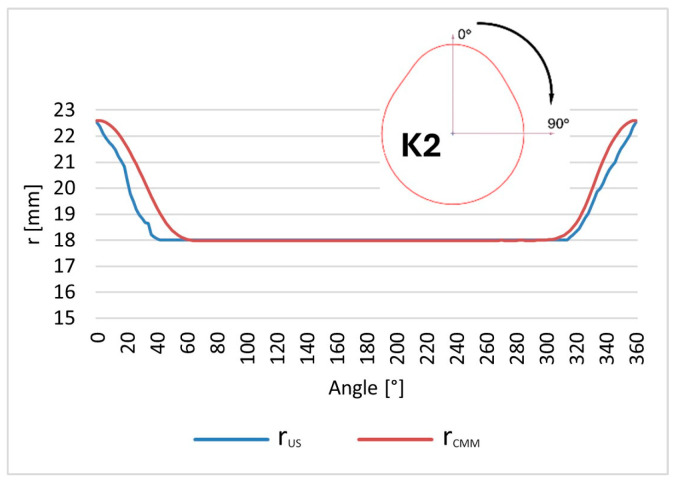
Comparison of the mean ${\bar{\Delta }}_{i}$ cam lobe K2 radius values measured by the robot with the reference values obtained from the coordinate measuring machine (CMM).

**Figure 13:**
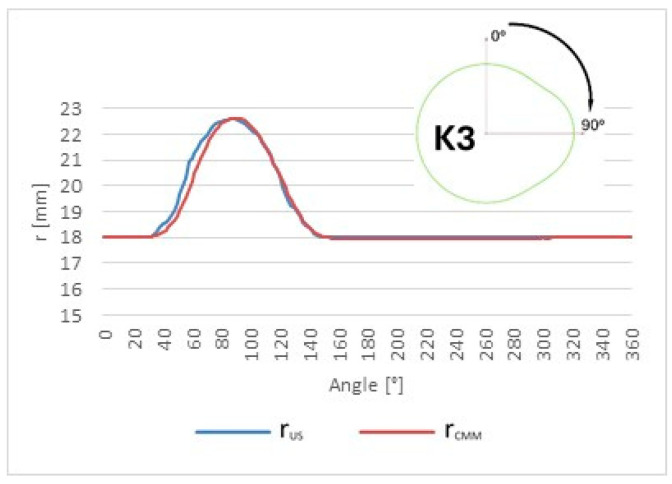
Comparison of the mean ${\bar{\Delta }}_{i}$ cam lobe K3 radius values measured by the robot with the reference values obtained from the coordinate measuring machine (CMM).

The value of ${\bar{\Delta }}_{i}$, representing the mean profile difference, enables evaluation of the average offset of the ultrasonic measurements relative to the reference CMM system. It can be interpreted as a measure of the systematic component of the measurement error of the entire measurement chain. The corresponding plots for each cam lobe (K1, K2, and K3) are presented in Figure 11, Figure 12 and Figure 13, respectively.

In addition, the local standard deviation of the differences $\sigma_{\Delta ,i}$ (7) was calculated for each angular position to quantify the random dispersion of ultrasonic measurements relative to the reference CMM value, thereby providing a measure of local measurement stability.

(7) $$\sigma_{\Delta ,i}\;=\;\sqrt{\frac{1}{n\;-\;1}\sum_{j=1}^{n}{\left[\left(r_{US,ij}\;-\;r_{CMM,i}\right)\;-\;{\bar{\Delta }}_{i}\right]}^{2}}$$

where:

$\sigma_{\Delta ,i}$―local standard deviation of the differences at the i-th angular position;

$r_{US,ij}$—the j-th ultrasonic measurement at the i-th sampling point;

${\bar{\Delta }}_{i}$―mean profile radius difference defined in (6).

**Figure 14:**
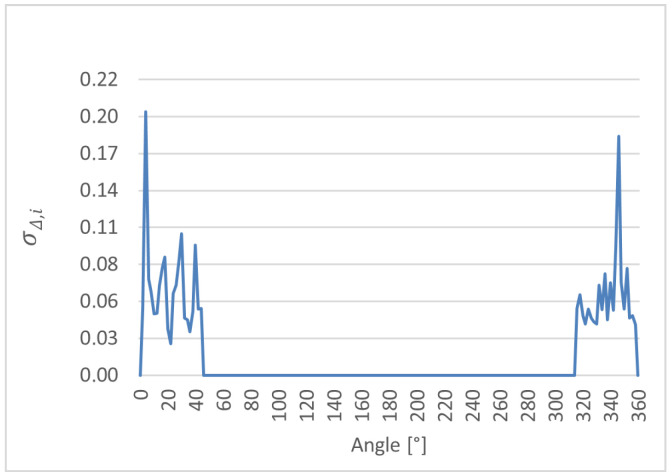
Local standard deviation of the differences ($\sigma_{\Delta ,i}$) as a function of angular position for cam lobe K1, obtained from 50 repeated ultrasonic measurements.

**Figure 15:**
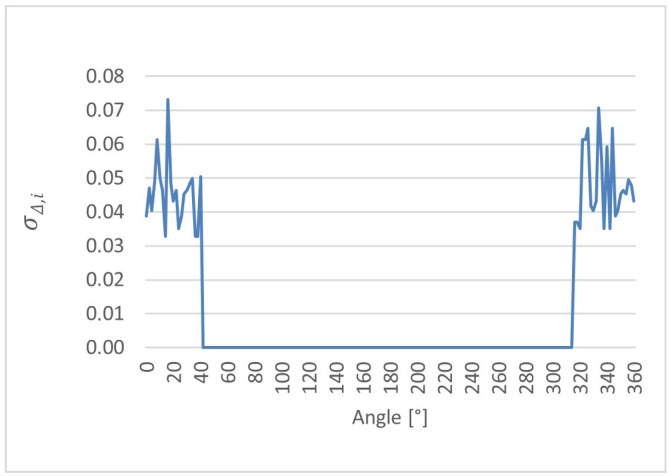
Local standard deviation of the differences ($\sigma_{\Delta ,i}$) as a function of angular position for cam lobe K2, obtained from 50 repeated ultrasonic measurements.

**Figure 16:**
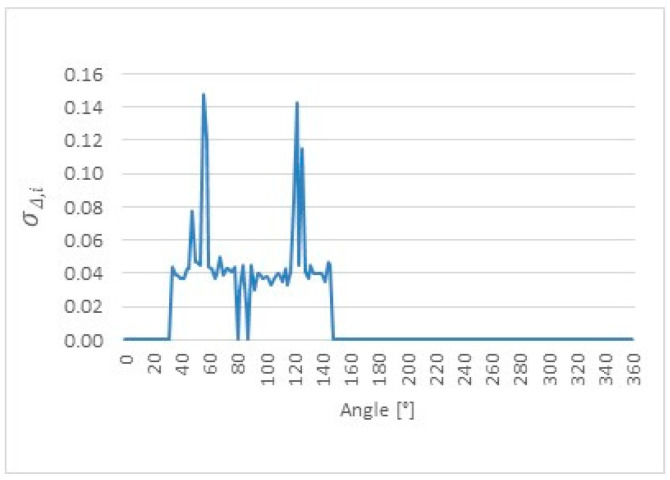
Local standard deviation of the differences ($\sigma_{\Delta ,i}$) as a function of angular position for cam lobe K3, obtained from 50 repeated ultrasonic measurements.

Based on the above definition, the local standard deviation of the differences $\sigma_{\Delta ,i}$ was evaluated for each angular position along the cam lobe profile. This analysis provides a pointwise assessment of the random dispersion of ultrasonic measurements relative to the reference CMM values and serves as a quantitative indicator of local measurement stability. The resulting distributions of $\sigma_{\Delta ,i}$ as a function of angular position are presented for cam lobes K1, K2, and K3 in Figure 14, Figure 15 and Figure 16, respectively. It should be noted that slight differences between repeated measurements may occur even at the same nominal angular position due to the sensitivity of the ultrasonic method to local surface curvature, sensor–surface orientation, and reflected echo stability. Therefore, repeated measurements were analyzed on a pointwise basis to assess local repeatability and geometry-dependent effects. Repetition of the complete 0–360° measurement cycle could provide additional insight into global measurement procedure stability and may be considered in future work.

Furthermore, the maximum absolute deviation was evaluated (8) locally for each angular position to identify the worst-case discrepancy between the ultrasonic measurements and the reference CMM value. For each angular position, the maximum absolute difference observed among all ultrasonic repetitions was determined.

(8) $${∆}_{max,i}\;=\;\max_{j=1,\ldots ,n}\left|r_{US,ij}\;-\;r_{CMM,i}\right|$$

where:

${∆}_{max,i}$―maximum local absolute deviation at the i-th angular position.

**Figure 17:**
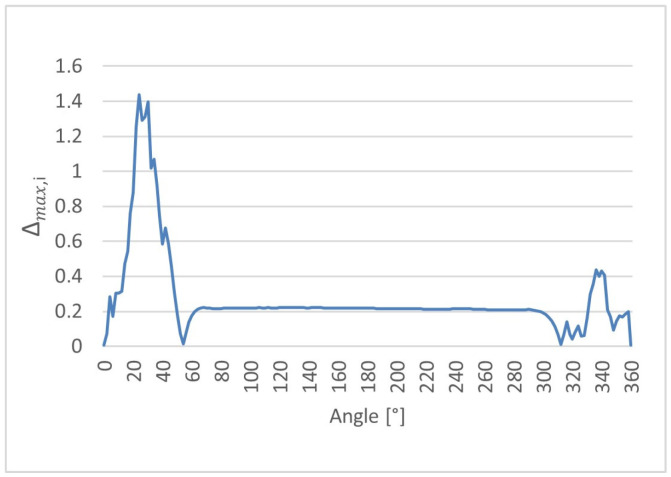
Local maximum absolute deviation (${∆}_{max,i}$) as a function of angular position for cam lobe K1, obtained from 50 repeated ultrasonic measurements.

**Figure 18:**
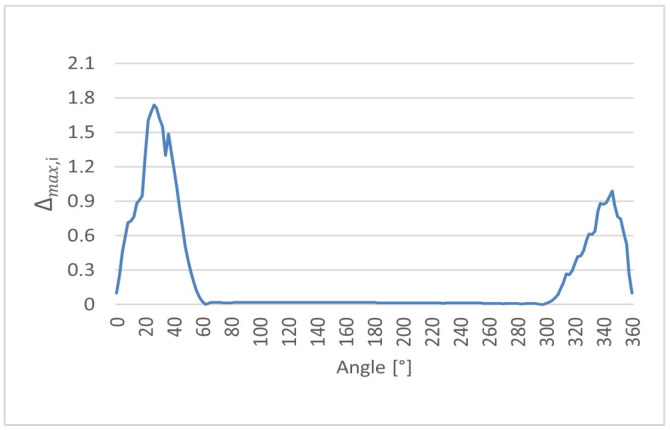
Local maximum absolute deviation (${∆}_{max,i}$) as a function of angular position for cam lobe K2, obtained from 50 repeated ultrasonic measurements.

**Figure 19:**
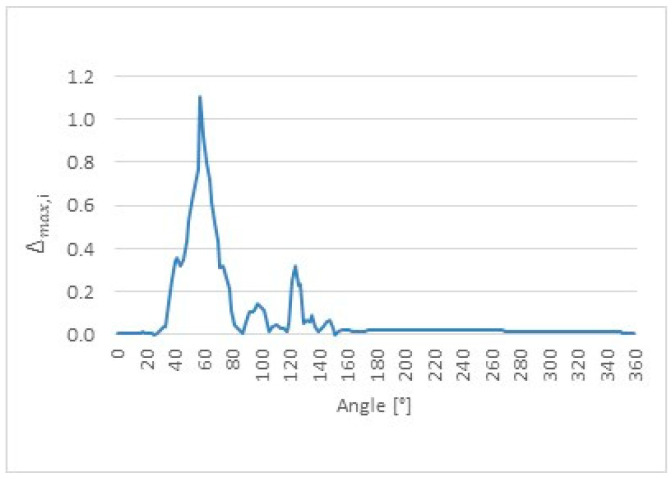
Local maximum absolute deviation (${∆}_{max,i}$) as a function of angular position for cam lobe K3, obtained from 50 repeated ultrasonic measurements.

The angular dependence of the local maximum absolute deviation ${∆}_{max,i}$ provides insight into the worst-case measurement discrepancies occurring along the cam lobe profile. The distributions of ${∆}_{max,i}$ as a function of angular position are presented for cam lobes K1, K2, and K3 in Figure 17, Figure 18 and Figure 19, respectively.

The comparative analysis of the cam lobe profiles was performed using pointwise error metrics evaluated at each angular position. The angular distributions of ${\bar{\Delta }}_{i}$, $\sigma_{\Delta ,i}$, and ${∆}_{max,i}$ were determined for cam lobes K1, K2, and K3. The results reveal a clear angular dependence of all metrics, with increased deviations observed in specific angular regions associated with characteristic geometric features of the cam lobes. These effects are consistently observed across all three cam lobes; however, their magnitude and angular extent vary depending on the local profile geometry.

## 6. Discussion

**Figure 20:**
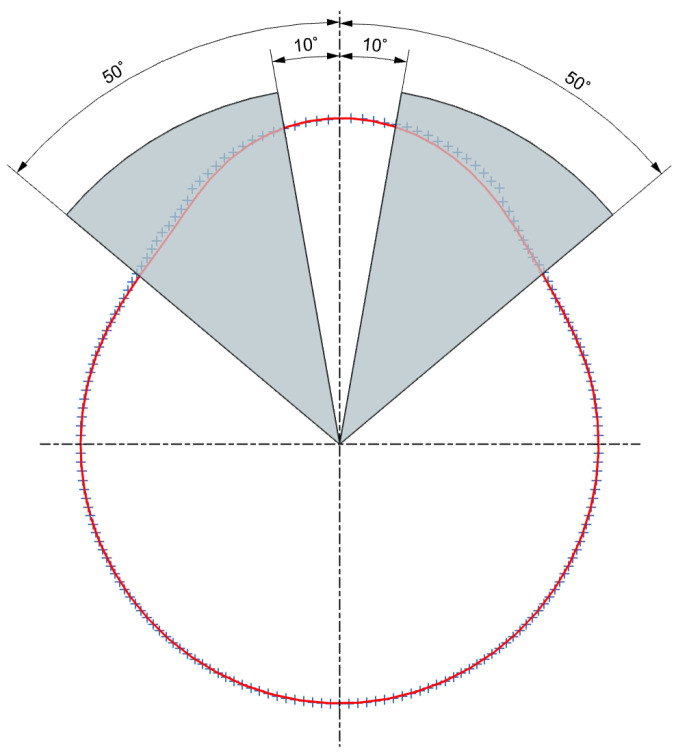
Geometric illustration of cam angular regions associated with increased ultrasonic measurement deviations.

The observed peaks in the local mean difference ${\bar{\Delta }}_{i}$ standard deviation $\sigma_{\Delta ,i}$, and maximum absolute deviation ${∆}_{max,i}$ are strongly correlated with the local geometric characteristics of the cam lobe profiles. In particular, increased deviations are consistently observed in angular regions corresponding to the rising and falling flanks of the cam profiles, where surface curvature changes rapidly and the local surface normal deviates significantly from the sensor axis, as illustrated in Figure 20.

The ultrasonic (US) distance sensor operates based on the time-of-flight principle. A short ultrasonic pulse is emitted toward the target surface, and the distance is determined from the time delay of the reflected echo. Measurement accuracy depends strongly on the orientation and local curvature of the measured surface, as these factors directly influence the amount of acoustic energy reflected to the receiver.

The angular variations observed in the mean profile radius difference ${\bar{\Delta }}_{i}$ the local standard deviation of the differences $\sigma_{\Delta ,i}$, and the local maximum absolute deviation ${∆}_{max,i}$ are consistent with the physical principles of ultrasonic wave propagation and reflection from curved surfaces. Increased values of ${\bar{\Delta }}_{i}$ indicate local systematic offsets, which arise when surface orientation and curvature reduce the reflected acoustic energy and introduce bias in the time-of-flight estimation.

Increased values of $\sigma_{\Delta ,i}$ are observed in angular regions where the local surface geometry changes rapidly. In these regions, the reflected echo becomes less stable, leading to increased variability in repeated ultrasonic measurements. Peaks in ${∆}_{max,i}$ represent worst-case discrepancies and are typically associated with occasional echo misdetections, such as multipath reflections or the selection of secondary echo peaks.

The occurrence of these deviations within specific angular intervals, as well as the differences observed among cam profiles K1, K2, and K3, reflects variations in local profile geometry and corresponding changes in surface normal orientation along each cam profile.

The similarity of these behaviors across all cam profiles indicates that the observed error patterns are primarily governed by geometric and acoustic interaction effects rather than random measurement noise. Differences in peak magnitude and angular extent between individual cam profiles can be attributed to variations in local curvature, flank length, and transition geometry.

These findings demonstrate that the measurement accuracy of ultrasonic sensing for camshaft inspection is strongly dependent on local surface geometry. This suggests that geometry-aware measurement strategies or multi-angle sensing approaches may significantly improve robustness in regions with challenging reflection conditions.

## 7. Conclusions

A prototype robotic workstation for cam lobe geometry measurement was developed and experimentally validated to achieve the preventive quality control of camshafts in industrial environments. The system combines a collaborative robot, dedicated fixturing, and an ultrasonic sensor, enabling the repeatable positioning of the measurement head and automated acquisition of cam profile data over the full angular range. The proposed indirect measurement model, together with the quadrant-based sensor orientation strategy and 90° camshaft indexing, enabled complete profile acquisition within the robot workspace.

Validation against coordinate measuring machine (CMM) reference measurements confirmed that the developed system provides repeatable measurement results, with a high level of agreement between the measured and reference cam profiles. However, pointwise error analysis revealed that the agreement between ultrasonic and CMM measurements is strongly dependent on angular position and local cam geometry. The largest deviations were observed in regions with rapidly changing curvature and unfavorable surface orientation relative to the ultrasonic beam.

Therefore, the proposed ultrasonic robotic system should be regarded primarily as a rapid preventive inspection tool for use on the production floor, rather than as a replacement for high-precision coordinate measuring machines. Its main advantages include automation of the inspection process, repeatable sensor positioning, suitability for rapid pre-assembly verification, and reduced operator dependency compared to manual inspection methods. In contrast, the CMM systems remain the preferred reference solution when maximum metrological accuracy is required.

The main limitation of the proposed method is its sensitivity to local surface curvature and sensor–surface orientation, which affects echo stability and leads to increased deviations in geometrically challenging regions. Future work will focus on improving measurement robustness through geometry-aware measurement planning, multi-angle sensing strategies, and potentially multi-sensor configurations. Further studies may also include extended industrial validation, cycle-time analysis, and evaluation of the economic benefits of implementing the system in production environments.

## Data Availability

Data is contained within the article.

## Abbreviations

The following abbreviations are used in this manuscript:

CMM	Coordinate Measuring Machine
ZDM	Zero-Defect Manufacturing
CAD	Computer-Aided Design
HMI	Human–Machine Interface
US	Ultrasonic
